# Effects of sedatives and opioids on trigger and cycling asynchronies throughout mechanical ventilation: an observational study in a large dataset from critically ill patients

**DOI:** 10.1186/s13054-019-2531-5

**Published:** 2019-07-05

**Authors:** Candelaria de Haro, Rudys Magrans, Josefina López-Aguilar, Jaume Montanyà, Enrico Lena, Carles Subirà, Sol Fernandez-Gonzalo, Gemma Gomà, Rafael Fernández, Guillermo M. Albaiceta, Yoanna Skrobik, Umberto Lucangelo, Gastón Murias, Ana Ochagavia, Robert M. Kacmarek, Montserrat Rue, Lluís Blanch, for the Asynchronies in the Intensive Care Unit (ASYNICU) Group, Candelaria de Haro, Josefina López-Aguilar, Rudys Magrans, Sol Fernández-Gonzalo, Gemma Gomà, Encarna Chacón, Anna Estruga, Ana Ochagavia, Lluís Blanch, Jaume Montanya, Bernat Sales, Enrico Lena, Umberto Lucangelo, Rafael Fernández, Carles Subirà, Guillermo M. Albaiceta, Enrique Fernández-Mondejar, Gastón Murias, Robert M. Kacmarek

**Affiliations:** 1 Critical Care Center, Parc Taulí Hospital Universitari, Institut d’Investigació i Innovació Parc Taulí I3PT, Universitat Autònoma de Barcelona, Sabadell, Spain; 2 Departament de Medicina, Universitat Autònoma de Barcelona, Barcelona, Spain; 3 CIBERES, Instituto de Salud Carlos III, Madrid, Spain; 4 Better Care, Barcelona, Spain; 5 Department of Perioperative Medicine, Intensive Care and Emergency, Cattinara Hospital, Trieste University, Trieste, Italy; 6 ICU, Fundació Althaia, Universitat Internacional de Catalunya, Manresa, Spain; 7 CIBERSAM, Instituto de Salud Carlos III, Madrid, Spain; 8 Unidad de Cuidados Intensivos Cardiológicos, Hospital Universitario Central de Asturias, Oviedo, Spain; 9 Departamento de Biología Funcional, Instituto Universitario de Oncología del Principado de Asturias, Universidad de Oviedo, Oviedo, Spain; 10 Department of Medicine, McGill University, Montréal, Québec Canada; 11 Regroupement des Soins Critiques Respiratoires, Réseau de Santé Respiratoire, Fonds de Recherche du Québec en Santé, Montréal, Québec Canada; 12 Critical Care Department, Hospital Británico, Buenos Aires, Argentina; 13 Department of Respiratory Care, Department of Anesthesiology, Massachusetts General Hospital, Harvard Medical School, Boston, MA USA; 14 Department of Basic Medical Sciences, Universitat de Lleida-IRB Lleida, Lleida, Spain; 15 Health Services Research Network in Chronic Diseases (REDISSEC), Madrid, Spain

**Keywords:** Asynchronies, Mechanical ventilation, Sedatives, Opioids, Double cycling, Ineffective inspiratory efforts during expiration

## Abstract

**Background:**

In critically ill patients, poor patient-ventilator interaction may worsen outcomes. Although sedatives are often administered to improve comfort and facilitate ventilation, they can be deleterious. Whether opioids improve asynchronies with fewer negative effects is unknown. We hypothesized that opioids alone would improve asynchronies and result in more wakeful patients than sedatives alone or sedatives-plus-opioids.

**Methods:**

This prospective multicenter observational trial enrolled critically ill adults mechanically ventilated (MV) > 24 h. We compared asynchronies and sedation depth in patients receiving sedatives, opioids, or both. We recorded sedation level and doses of sedatives and opioids. BetterCare™ software continuously registered ineffective inspiratory efforts during expiration (IEE), double cycling (DC), and asynchrony index (AI) as well as MV modes. All variables were averaged per day. We used linear mixed-effects models to analyze the relationships between asynchronies, sedation level, and sedative and opioid doses.

**Results:**

In 79 patients, 14,166,469 breaths were recorded during 579 days of MV. Overall asynchronies were not significantly different in days classified as sedatives-only, opioids-only, and sedatives-plus-opioids and were more prevalent in days classified as no-drugs than in those classified as sedatives-plus-opioids, irrespective of the ventilatory mode. Sedative doses were associated with sedation level and with reduced DC (*p* < 0.0001) in sedatives-only days. However, on days classified as sedatives-plus-opioids, higher sedative doses and deeper sedation had more IEE (*p* < 0.0001) and higher AI (*p* = 0.0004). Opioid dosing was inversely associated with overall asynchronies (*p* < 0.001) without worsening sedation levels into morbid ranges.

**Conclusions:**

Sedatives, whether alone or combined with opioids, do not result in better patient-ventilator interaction than opioids alone, in any ventilatory mode. Higher opioid dose (alone or with sedatives) was associated with lower AI without depressing consciousness. Higher sedative doses administered alone were associated only with less DC.

**Trial registration:**

ClinicalTrial.gov, NCT03451461

**Electronic supplementary material:**

The online version of this article (10.1186/s13054-019-2531-5) contains supplementary material, which is available to authorized users.

## Background

Patient-ventilator asynchronies are frequent during invasive mechanical ventilation (MV) [1, 2]. Poor patient-ventilator interaction could be associated with prolongation of MV, longer intensive care unit (ICU) and hospital stays [2], and increased mortality [1, 3]. Thus, optimizing patient-ventilator interaction may improve outcomes [4].

Many aspects of clinical management affect patient-ventilator interaction. Adjusting ventilator settings can decrease asynchronies and associated anxiety and dyspnea [5, 6]. Sedatives can cause ventilatory depression affecting respiratory drive and timing, worsening patient-ventilator interaction in proportion to decreasing level of consciousness [7, 8]; these effects appear to differ with different drugs [9, 10]. Sedation is associated with deleterious side effects. Deep sedation is associated with worse short- and long-term outcomes [11–14]. Forgoing or minimizing sedatives during MV is increasingly recommended [15–17].

The relationship between asynchronies, level of consciousness (sedation level), and sedatives and opioids is poorly understood. Increasing sedatives in ICU patients with double cycling (DC) not only failed to correct this asynchrony [6], but also prolonged MV and ICU and hospital stays. The rate of ineffective inspiratory efforts increases proportionately with the depth of sedation [8]. Different studies have reported disparate effects of sedative dosage on the overall rate of asynchronies. Whereas one study found that light sedation with propofol did not affect the rate of asynchronies but deep sedation with propofol increased it [10], another found that deep sedation reduced but did not eliminate asynchronies [18]. A recent study showed that deep sedation, benzodiazepines, and cumulative doses of benzodiazepines were associated with higher mortality [19]. In another trial, patients on dexmedetomidine had slightly fewer asynchronies than those on propofol [20]. However, these studies did not take opioid administration into account.

Opioids are commonly used in ICU patients. In a study comparing midazolam vs. fentanyl plus midazolam, patients receiving fentanyl had fewer asynchronies than those receiving only midazolam [21]. Thus, the effects of opioids alone or together with sedatives on asynchronies warrant investigation. Adequate opioid treatment with minimal doses of sedatives might enable more spontaneous breathing and improve patient-ventilator interaction; however, the relationships between opioids, level of consciousness, and asynchronies remain to be elucidated. We hypothesized that opioids alone would improve trigger and cycling asynchronies and result in more wakeful patients than sedatives alone or sedatives-plus-opioids.

## Methods

### Study population and design

We obtained data from an ongoing database started in 2011 in four centers in Spain. The database was constructed prospectively with funding for a project to develop a connectivity platform to interoperate signals from different ventilators and monitors and subsequently compute algorithms to diagnose patient-ventilator asynchronies (ClinicalTrial.gov, NCT03451461); each institution’s review board approved the database.

This prospective observational study included adult patients admitted to four ICUs between October 2011 and January 2013. The institutional review boards approved the protocol, waiving informed consent because the study was non-interventional, posed no added risk to patients, and did not interfere with usual care.

Patients were prospectively included when the following criteria were met: admission to a bed equipped with BetterCare™ software and intubated for MV expected to last > 24 h. To avoid selection bias, members of the research team were not involved in assigning patients to a bed equipped with BetterCare™ software. Exclusion criteria were < 48 h of data, age < 18 years, pregnancy, do-not-resuscitate orders, admission for organ donation, and chest tubes with suspected bronchopleural fistula.

### Patient management and data collection

Demographic and clinical data were obtained from medical records. Level of consciousness was assessed every 4 h with the Riker Sedation-Agitation Scale (SAS), and the mean value of these assessments was computed to obtain a daily average. Illness severity was assessed daily with the Sequential Organ Failure Assessment (SOFA). ICU teams were aware of the recording system, but not of the study hypothesis. All patients were managed with similar processes of care and lung-protective ventilation strategies (tidal volume 6 mL/kg ideal body weight and plateau pressure under 30 cmH_2_O) following the quality indicators of the Spanish Society of Intensive Care Medicine (https://semicyuc.org/wp-content/uploads/2018/10/quality_indicators_update_2011.pdf) throughout the study. Patients were ventilated with Evita 4 (Dräger, Lübeck, Germany), Puritan Bennet 840 (Covidien, Plymouth, MN, USA), or Servo I (Maquet, Fairfield, NJ, Sweden) ventilators, receiving volume assist/control, pressure assist control, or pressure support based on clinicians’ assessment of clinical status. Ventilatory modes were analyzed as previously described [22]. The predominant mode for each day analyzed was classified as assist-control or pressure-support if the patient remained in that mode for ≥ 70% of the time. Other ventilator parameters were also adjusted at the discretion of the attending physician following national recommendations. Clinicians adjusted ventilator settings when asynchronies were observed at the bedside, but adjustments were not protocolized.

Total doses of opioids (morphine and fentanyl) and sedatives (midazolam, propofol, and lorazepam) administered each day were recorded and converted to morphine and midazolam equivalents [6]. We classified each day of MV for a given patient as (1) no-drugs, (2) sedatives-only, (3) opioids-only, or (4) sedatives-plus-opioids. To avoid misleading classifications due to residual treatment effects, days were classified according to all medications administered during the day; thus, a day in which a patient received sedatives-plus-opioids for > 2 h and opioids-only thereafter would be classified as “sedatives-plus-opioids.” Days in which patients were treated with neuromuscular blockers were excluded from the analysis. Sedatives and analgesia were managed following each ICU’s protocols based on the Spanish Society of Intensive Care Medicine recommendations [23], SAS level, and pain and discomfort assessments.

For the analyses, data was structured as averaged measures per day. Therefore, every treatment group could include a different number of patients depending on the day.

### Analysis of asynchronies

Asynchronies were detected by BetterCare™ software (Barcelona, Spain), which continuously records airflow, airway pressure, and tidal volume from admission to extubation or death. BetterCare™ identifies the beginnings of inspiration and expiration to analyze and store data breath by breath. It analyzes each breath to detect four types of asynchronies (ineffective inspiratory efforts during expiration (IEE), DC, short cycling/aborted inspiration, and prolonged cycling [1, 2]) and computes the asynchrony index (AI) (Additional file 1) [24]. Periods in which recording was interrupted due to clinical interventions, out-of-ICU transfers, technical problems, or other issues were excluded from the analysis, which was done on the remaining valid periods.

All asynchronies were averaged per day. The rates of IEE, DC, and the overall AI were computed considering the total number of breaths (ventilator-delivered cycles plus IEE), enabling us to compare days, despite varying respiratory rates.

### Statistical analysis

Patients’ characteristics are summarized as medians (25th–75th percentiles) or percentages. Sample size calculation was considered unnecessary for this exploratory study.

To analyze the level of consciousness and illness severity by treatment groups, we used linear mixed-effects (LME) models with random intercepts for the patients. This approach takes inter- and intra-subject variability in longitudinal data into account; each patient differs from the overall mean response by an individual-specific constant that applies equally over time [25]. To analyze asynchronies by treatment groups, we used generalized LME (GLME) models assuming a negative binomial distribution for the response variable (number of asynchrony events) because the variable response was discrete, limited to non-negative values, and positively skewed with most observations having values near zero. Negative binomial distributions are often used in regression models with count data. Furthermore, to analyze the number of asynchrony events as a rate, we incorporated an exposure term (total number of respiratory cycles per day) that indicates the number of times a particular event occurred.

To assess the effects of level of consciousness and severity of illness on each type of asynchrony, we also used GLME models with random intercepts for the patients and allowing variation in SAS and SOFA slopes by treatment groups for the population mean.

To explore the effects of dosage on level of consciousness and asynchronies, we used a LME model and GLME models, respectively. This analysis included the opioids-only, sedatives-only, and sedatives-plus-opioids groups. These models used random intercepts only for the patients and allowed variations in slopes for dose equivalents by treatment groups for the population mean. Additionally, we investigated the effect of severity (SOFA) as a potential confounding variable that could influence both the asynchronies and the treatment group.

We used R 3.3.1 (R Core Team, Vienna, Austria, URL http://www.R-project.org) for all analyses, building the mixed-effects models with the lme4 package [25] and summarizing the mean (95% CI) effects by treatment groups with the lsmeans package. When using LME models for the continuous response variables, we checked the normality assumptions for the estimated random effects and for the within-subject residuals by graphical methods (normal Q-Q plots). When the response variable was discrete, we assessed overdispersion by graphical comparison of the standardized residuals versus the fitted values. Significance was set at *p* < 0.01. Pairwise comparisons among treatment groups were two-sided and adjusted by the Bonferroni method to maintain the significance level.

## Results

Table 1 reports on the demographic, clinical, and outcome data for the 79 patients. We analyzed 579 days on invasive MV, comprising 14,166,469 breaths.

**Table 1:** Patients’ demographic and clinical characteristics

Total population (*n* = 79)	Median [25th, 75th percentiles]	Percentage
Age (years)	63 [52, 75]	
Sex (% men)		64.5%
Reason for admission *n*		
Acute respiratory failure		39 (49.4%)
- Sepsis		12 (15.2%)
- Pneumonia		7 (8.7%)
- ARDS		5 (6.3%)
- COPD		3 (3.8%)
- Congestive heart failure		2 (2.5%)
- Other		10 (12.7%)
Neurologic		15 (19%)
Cardiac arrest		10 (12.7%)
Postsurgical		8 (10.1%)
Multiple trauma		6 (7.6%)
Neuromuscular disease		1 (1.3%)
APACHE II	17 [10, 26]	
SOFA at admission	7 [5.25, 10.75]	
Length of mechanical ventilation (days)	6 [3, 10.5]	
ICU stay (days)	10 [6, 18]	
Hospital stay (days)	23 [11, 50]	
Mortality ICU		27.9%

*ARDS* acute respiratory distress syndrome, *COPD* chronic obstructive pulmonary disease, *APACHE* Acute Physiology and Chronic Health Evaluation, *SOFA* Sequential Organ Failure Assessment score, *ICU* intensive care unit

### Relationship between asynchronies and treatment group

**Fig. 1:**
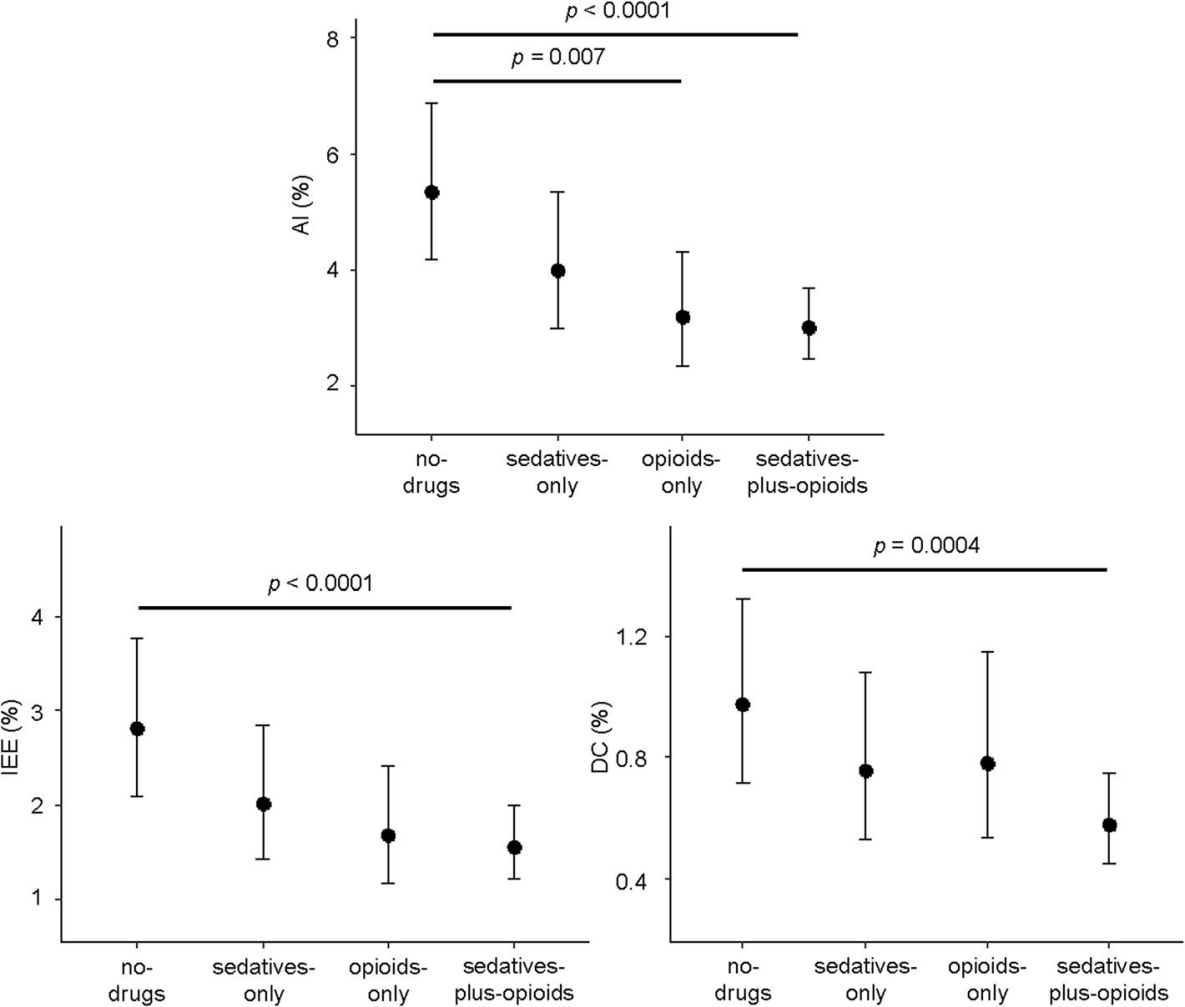
Mean percentages of asynchronous breaths estimated with the generalized linear mixed-effects model by treatment groups. Data are represented as mean (95% CI). Statistical significance (two-sided) among groups is indicated; *p* values are adjusted by the Bonferroni method

Figure 1 shows the relationship between each asynchrony and treatment group. No statistically significant differences in AI, IEE, or DC were found between sedatives-only, opioids-only, and sedatives-plus-opioids days. The AI and rates of IEE and DC were higher for no-drugs days than for sedatives-plus-opioids days; AI was also higher for no-drugs than for opioids-only days.

### Relationships between treatment group, level of consciousness, and severity of illness

**Fig. 2:**
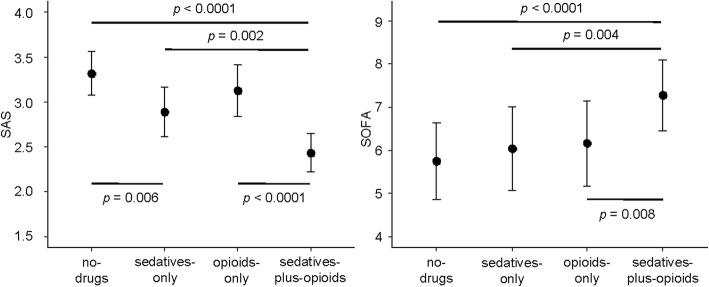
Mean levels of SAS and SOFA estimated with the linear mixed-effects model by treatment groups. Data are represented as mean (95% CI). Statistical significance (two-sided) among groups is indicated; *p* values are adjusted by the Bonferroni method. The within-subject residuals of the SOFA model departure from the theoretical normal distribution (see Additional file 5: Figure S4 left)

Mean daily SAS in sedatives-plus-opioids days [2.4 (95%CI 2.2–2.6)] was lower than in opioids-only [3.1 (95%CI 2.8–3.4); *p* < 0.0001], sedatives-only [2.9 (95%CI 2.6–3.2); *p* = 0.002], and no-drugs days [3.3 (95%CI 3.1–3.6); *p* < 0.0001]. Mean daily SAS in no-drugs days was higher than in sedatives-only days (*p* = 0.006) (Fig. 2). SOFA scores in sedatives-plus-opioids days were higher than in no-drugs (*p* < 0.0001), sedatives-only (*p* = 0.004), and opioids-only days (*p* = 0.008); SOFA scores were similar in the no-drugs, sedatives-only, and opioids-only groups (Fig. 2).

Table 2 summarizes the relationships of the level of consciousness and severity with asynchronies in the treatment groups. Higher level of consciousness was associated with higher DC rates (*p* < 0.0001) in sedatives-plus-opioids and sedatives-only days (*p* < 0.0001). However, the level of consciousness was not associated with AI or IEE regardless of exposure to opioids or sedatives. SOFA was not associated with AI or IEE, but was associated with a higher DC rate in no-drugs days (*p* = 0.008).

**Table 2:** Mean estimated effect from the regression coefficient of SAS and SOFA on asynchronies, by treatment group

Treatment group	Asynchrony Index	Ineffective inspiratory efforts during expiration	Double cycling
SAS			
No drugs	− 0.10 (− 0.29, 0.10) *p* = 0.34	− 0.14 (− 0.36, 0.09) *p* = 0.24	− 0.02 (− 0.25, 0.21) *p* = 0.87
Sedatives	0.11 (− 0.09, 0.31) *p* = 0.29	− 0.04 (− 0.27, 0.20) *p* = 0.76	0.46 (0.23, 0.69) *p* < 0.0001
Opioids	− 0.17 (− 0.37, 0.04) *p* = 0.12	− 0.20 (− 0.44, 0.04) *p* = 0.10	0.08 (− 0.18, 0.33) *p* = 0.55
Sedatives + opioids	0.14 (0.03, 0.26) *p* = 0.17	0.12 (− 0.02, 0.26) *p* = 0.09	0.30 (0.17, 0.44) *p* < 0.0001
SOFA			
No drugs	0.02 (− 0.03, 0.07) *p* = 0.38	0.02 (− 0.04, 0.08) *p* = 0.52	0.08 (0.02, 0.13) *p* = 0.008
Sedatives	0.02 (− 0.05, 0.09) *p* = 0.55	0.06 (− 0.02, 0.14) *p* = 0.17	− 0.03 (− 0.12, 0.05) *p* = 0.45
Opioids	− 0.06 (− 0.13, 0.02) *p* = 0.13	− 0.05 (− 0.13, 0.03) *p* = 0.25	− 0.09 (− 0.17, − 0.01) *p* = 0.03
Sedatives + opioids	− 0.01 (− 0.05, 0.03) *p* = 0.66	− 0.00 (− 0.05, 0.05) *p* = 0.98	− 0.00 (− 0.05, 0.04) *p* = 0.91

Results are expressed as mean estimated effect and 95% CI. A negative sign indicates an inverse association. Statistically significant associations are indicated

*SAS* Sedation Assessment Scale, *SOFA* Sequential Organ Failure Assessment

### Relationship between asynchronies and drug doses

**Fig. 3:**
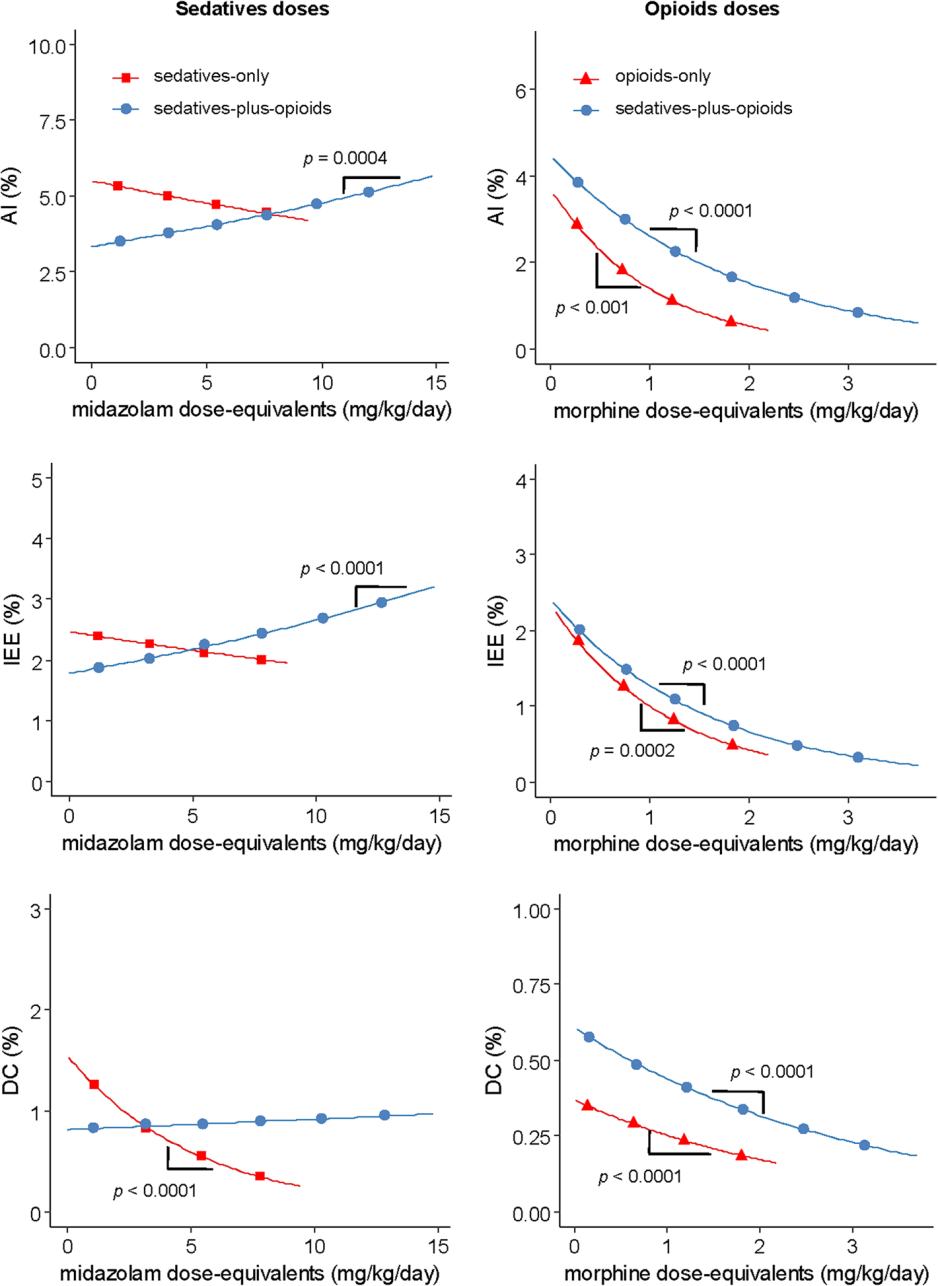
Effect of the dose of sedatives and opioids administered on asynchronies. Average change in asynchronies per one unit change in dose equivalent

Figure 3 illustrates the relationship between each asynchrony and doses of sedatives (left) and opioids (right). In opioids-only days (red, right panel, Fig. 3), the opioid dose was inversely associated with AI (*p* < 0.001), IEE (*p* = 0.0002), and DC (*p* < 0.0001). In sedatives-plus-opioids days (blue, right panel, Fig. 3), opioid dose was also inversely associated with AI, IEE, and DC (*p* < 0.0001), whereas sedative dose was directly associated with AI (*p* = 0.0004) and IEE (*p* < 0.0001), but not with DC (in blue, left panel Fig. 3). However, in sedatives-only days (red trace, left panel Fig. 3), sedative doses were inversely associated only with DC (*p* < 0.0001). Additional file 2: Table S1 reports on the regression coefficients and performance of the model examining the relationship between medication dose and asynchronies.

When SOFA score was included as a potential confounding factor (Additional file 2: Table S2), the direction of the above statistically significant associations remained unchanged, except in the opioids-only for the DC model, where the association was no longer significant. In addition, the SOFA was not associated with any of the asynchrony variables.

### Relationship between drug dose and level of consciousness

Additional file 3: Figure S2 shows the relationship between the level of consciousness and doses of sedatives (left panel) and of opioids (right panel). Higher sedative doses were associated with a lower level of consciousness, in both sedatives-only (*p* < 0.0001; red, left panel) and sedatives-plus-opioids days (*p* = 0.004; blue, left panel). Opioid doses were not associated with the level of consciousness.

### Relationship between asynchronies, treatment group, and mechanical ventilation modes

**Fig. 4:**
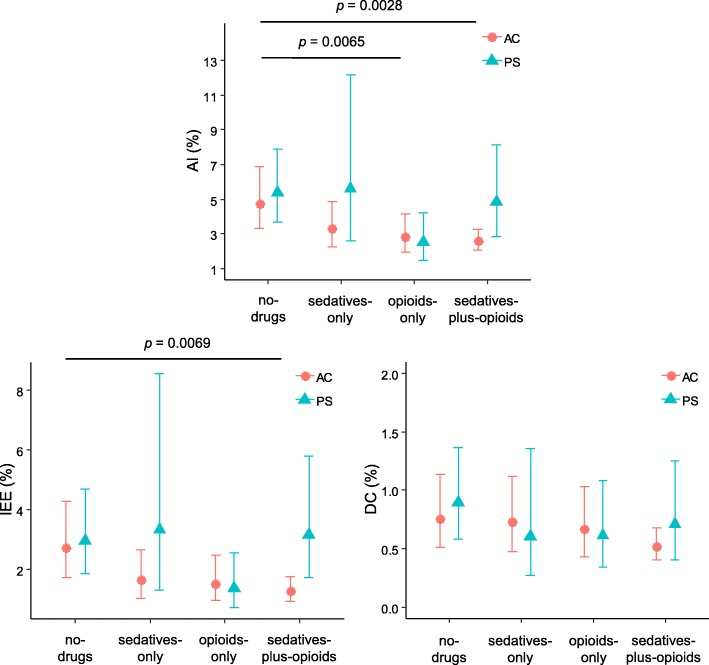
Mean percentages of asynchronous breaths estimated with the generalized linear mixed-effects model according to mechanical ventilator mode, by treatment groups. Data are represented as mean (95% CI)

We analyzed the effect of MV modes in the incidence of asynchronies in each treatment group. There were no statistically significant differences in the AI, IEE, or DC between assist-control and pressure support modes in any treatment group (*p* > 0.01). In assist-control mode, the opioids-only and sedatives-plus-opioids groups had a lower AI than the no-drugs group (*p* = 0.0065 and *p* = 0.0028, respectively) (Fig. 4) (Additional file 4: Table S3).

## Discussion

This is the first study to present data relating asynchronies and treatment with sedatives and opioids throughout the complete MV period. The overall rate of asynchronies did not differ between days classified as opioids-only, sedatives-only, and sedatives-plus-opioids. Patients receiving sedatives had a lower level of consciousness than those receiving opioids-only; sedatives-plus-opioids decreased the level of consciousness, but did not result in fewer asynchronies than the other treatments. Interestingly, in sedatives-plus-opioids days, the sedative dose was directly associated with the rate of asynchronies and with a lower level of consciousness, whereas higher opioid doses were associated with a lower AI without worsening level of consciousness. Thus, opioid administration seems a clinically sound approach to improve patient-ventilator synchrony while preserving consciousness.

Patients with shock or severe respiratory failure often require sedatives. Moreover, sedatives are sometimes administered in attempts to improve patient-ventilator interaction. However, deep sedation has been associated with worse outcomes [13]. Lighter or no-sedation is favored in partial ventilatory support modes, where patient-ventilator synchrony is crucial. Physiological studies show that sedatives have varying effects on asynchronies. Increasing sedatives/analgesia is relatively ineffective in abolishing severe breath-stacking [6]. Deep sedation with propofol increases asynchronies during pressure support ventilation, whereas light sedation does not [10]; deeper sedation is associated with increased ineffective triggering events [8] and with increased mortality [26]. Thus, sedative management in MV is a modifiable variable that could improve outcomes [26].

Associations between sedatives and asynchronies are probably confounded by many factors, especially by clinicians’ ventilator adjustments. In our study, the incidence of asynchronies was associated with the drugs used (sedatives and opioids), irrespective of the ventilatory mode. However, sedatives lowered patients’ level of consciousness without decreasing asynchronies beyond opioids alone. Inadequate pain control worsens patient-ventilator synchrony [21] and is associated with agitation, which negatively affects outcomes [27]. Richman et al. [21] found that patients receiving midazolam-plus-opioids had fewer asynchronies/day over a 3-day period than those receiving midazolam alone. Our results support these findings, showing that opioids could help improve asynchronies beyond sedatives, although prospective trials are necessary to determine whether appropriate opioids favor better synchrony by ensuring adequate analgesia without depressing consciousness and without affecting respiratory drive or minute ventilation [28–30].

Increasing sedative doses prolongs MV and ICU and hospital stays [12, 14]. Paired with pain management protocols [17], sedation protocols including light sedation or daily interruptions of sedation [13, 15, 16] improve ICU patients’ outcomes. Our results suggest that, compared with treatments including sedatives, treatment with opioids-only enables patients to be more awake without increasing asynchronies. Opioids-only treatment resulted in a higher level of consciousness than treatment with sedatives-plus-opioids. Our findings on the effects of sedatives and opioids throughout MV are in line with those of a randomized clinical trial where MV patients receiving morphine had more ventilator-free days and shorter ICU stays than those receiving sedatives-plus-morphine, without increases in accidental extubation or ventilator-associated pneumonia [16]. Moreover, we found that in assist-control modes, compared with no-drugs, treatment with sedatives-plus-opioids and opioids-only favored better patient-ventilation interaction (lower AI), suggesting that opioids might improve patient comfort. Thus, it might be beneficial to maintain opioid treatment until liberation from MV.

We also found significantly lower AI, IEE, and DC in sedatives-plus-opioids than in no-drugs days. However, asynchronies in MV patients who do not require sedatives or opioids probably are intrinsically different from those that occur in patients who require these treatments, and therefore, they probably require a different clinical approach. Rue et al. [31, 32] recently used a Bayesian joint model incorporating longitudinal ICU stay markers to evaluate outcome determinants. Finding that overall AI was not associated with severity of illness or vital status, they postulated that some asynchronies could be a marker of life. In our study, we considered the effect of severity of illness to better understand this possible confounding factor. We found no associations between severity and AI or IEE (Table 2), so patients with more asynchronies were not necessarily more severely ill. The lower severity and higher rates of asynchronies in no-drugs days compared with sedatives-only, opioids-only, and sedatives-plus-opioids days are likely due to differences in the origin and behavior of the asynchronies that occur in the different groups. Therefore, from a clinical perspective, it makes no sense to compare asynchronies and their management in no-drugs versus in treatment days [3, 33].

Finally, our analysis of the relationship between asynchronies and drug dosage found that, unlike sedatives, increasing doses of opioids were associated with decreasing rates of asynchronies, without significantly affecting the level of consciousness, independently of the level of severity. This finding highlights the importance of titrating opioids for comfort, and possibly asynchronies, in addition to pain control.

Unlike some physiological studies, we found that sedatives, alone or together with opioids, did not decrease asynchronies more than opioids-only. On the other hand, our findings of increased rates of asynchronies, especially IEE, with an increased daily dose of sedatives are in line with physiological studies [8, 10]. Whereas physiological studies analyze only short time periods, our study considered the entire period of MV, making it closer to clinical practice. One strength of our study is that it is based on prospectively accrued physiological data with enough breadth and depth to characterize a patient’s condition throughout MV. Observational trials like this are increasingly being used because conclusions drawn from data collected in real-world situations can be more generalizable than the more restricted, if more vigorous, conclusions of randomized clinical trials [34–36]. Our findings add to the growing body of evidence pointing to the inability of sedatives to prevent and/or correct asynchronies in daily practice and the association of opioids with improved asynchrony rates, thus supporting the strategy of managing pain while maintaining the lightest sedation possible [17, 37].

Our study has several limitations. First, patients were not randomized to each drug regimen. Furthermore, patients received opioids, sedatives, or no-drugs in any sequence or combination as deemed clinically necessary. Granular data for sedative or opioid doses over smaller time intervals were unavailable, thus precluding analyses of temporal associations between sedatives/opioids and asynchronies that might have enabled causal inferences and greater insight. Moreover, we did not consider factors that influenced clinicians to modify sedative or opioid dosing. Patients often receive more drugs early in MV and less when approaching weaning; however, to counterbalance this bias, we explored the relationship between severity and drugs and asynchronies, but found no relevant associations. Additionally, we did not analyze other painkillers such as acetaminophen and nonsteroidal anti-inflammatory drugs, which may influence patient-ventilator interaction differently, so our findings cannot be extrapolated to other non-opioid drugs. We used no objective measures of pain levels, precluding the analysis of associations between pain and asynchronies. Likewise, we did not measure surrogates of respiratory center activity, so we cannot evaluate associations between respiratory drive and different asynchronies. As individual patients could be considered in more than one group because management strategies evolved from day-to-day, our analysis of drug doses could not take into account the prolonged half-life and accumulation of some sedatives [38]. Nevertheless, we found a good relationship between SAS score and the sedative treatment group. We analyzed only IEE and DC because they are the most relevant asynchronies; we did not analyze flow asynchronies because they cannot be established from ventilator airway pressure and flow scalars alone. Thus, our findings cannot be extrapolated to these asynchronies. Additionally, because the measure unit was days rather than patients, it was difficult to analyze the effect of the underlying disease on the results. In an attempt to overcome this difficulty, we adjusted the results by including SOFA score as a marker of severity, but we found no effect of severity in the incidence of asynchronies per group. Finally, clinically detected asynchronies were treated according to each ICU’s protocols; thus, differences between centers might have affected the results. We performed an analysis to evaluate the influence of each center and we did not find significant differences between centers.

## Conclusions

Our findings suggest that sedatives, alone or together with opioids, do not decrease asynchronies beyond what can be achieved with opioids alone, independently of MV mode. Optimal titration of opioids might improve patient-ventilator interaction while avoiding the deleterious effects of sedatives.

## Additional files

Additional file 1: Patient-ventilator asynchronies. (DOCX 278 kb)

Additional file 2: Asynchronies and medication dose and asynchronies and medication dose plus SOFA as a potential confounding variable. (DOCX 86 kb)

Additional file 3: Sedatives and opioids dose and level of consciousness. (DOCX 68 kb)

Additional file 4: Asynchronies and treatment group plus mechanical ventilation mode. (DOCX 78 kb)

Additional file 5: Checking assumptions of the (generalized) linear mixed-effects models. (DOCX 369 kb)

## Data Availability

The datasets used and analyzed during this study are available from the corresponding author on reasonable request.

## Abbreviations

AI: Asynchrony index
DC: Double cycling
ICU: Intensive care unit
IEE: Inspiratory efforts during expiration
MV: Mechanical ventilation
SAS: Riker Sedation-Agitation Scale
SOFA: Sequential Organ Failure Assessment

## References

[1] Blanch L, Villagra A, Sales B, Montanya J, Lucangelo U, Lujan M, Garcia-Esquirol O, Chacon E, Estruga A, Oliva JC (2015). Asynchronies during mechanical ventilation are associated with mortality. Intensive Care Med.

[2] Thille AW, Rodriguez P, Cabello B, Lellouche F, Brochard L (2006). Patient-ventilator asynchrony during assisted mechanical ventilation. Intensive Care Med.

[3] Vaporidi K, Babalis D, Chytas A, Lilitsis E, Kondili E, Amargianitakis V, Chouvarda I, Maglaveras N, Georgopoulos D (2017). Clusters of ineffective efforts during mechanical ventilation: impact on outcome. Intensive Care Med.

[4] Subira C, de Haro C, Magrans R, Fernandez R, Blanch L (2018). Minimizing asynchronies in mechanical ventilation: current and future trends. Respir Care.

[5] Schmidt M, Demoule A, Polito A, Porchet R, Aboab J, Siami S, Morelot-Panzini C, Similowski T, Sharshar T (2011). Dyspnea in mechanically ventilated critically ill patients. Crit Care Med.

[6] Chanques G, Kress JP, Pohlman A, Patel S, Poston J, Jaber S, Hall JB (2013). Impact of ventilator adjustment and sedation-analgesia practices on severe asynchrony in patients ventilated in assist-control mode. Crit Care Med.

[7] Bassuoni AS, Elgebaly AS, Eldabaa AA, Elhafz AA (2012). Patient-ventilator asynchrony during daily interruption of sedation versus no sedation protocol. Anesth Essays Res.

[8] de Wit M, Pedram S, Best AM, Epstein SK (2009). Observational study of patient-ventilator asynchrony and relationship to sedation level. J Crit Care.

[9] Morel DR, Forster A, Bachmann M, Suter PM (1984). Effect of intravenous midazolam on breathing pattern and chest wall mechanics in human. J Appl Physiol Respir Environ Exerc Physiol.

[10] Vaschetto R, Cammarota G, Colombo D, Longhini F, Grossi F, Giovanniello A, Della Corte F, Navalesi P (2014). Effects of propofol on patient-ventilator synchrony and interaction during pressure support ventilation and neurally adjusted ventilatory assist. Crit Care Med.

[11] Fernandez-Gonzalo S., Turon M., De Haro C., López-Aguilar J., Jodar M., Blanch L. (2018). Do sedation and analgesia contribute to long-term cognitive dysfunction in critical care survivors?. Medicina Intensiva (English Edition).

[12] Jarman A, Duke G, Reade M, Casamento A (2013). The association between sedation practices and duration of mechanical ventilation in intensive care. Anaesth Intensive Care.

[13] Shehabi Y, Chan L, Kadiman S, Alias A, Ismail WN, Tan MA, Khoo TM, Ali SB, Saman MA, Shaltut A (2013). Sedation depth and long-term mortality in mechanically ventilated critically ill adults: a prospective longitudinal multicentre cohort study. Intensive Care Med.

[14] Zhu Y, Wang Y, Du B, Xi X (2017). Could remifentanil reduce duration of mechanical ventilation in comparison with other opioids for mechanically ventilated patients? A systematic review and meta-analysis. Crit Care.

[15] Kress JP, Pohlman AS, O'Connor MF, Hall JB (2000). Daily interruption of sedative infusions in critically ill patients undergoing mechanical ventilation. N Engl J Med.

[16] Strom T, Martinussen T, Toft P (2010). A protocol of no sedation for critically ill patients receiving mechanical ventilation: a randomised trial. Lancet.

[17] Barr J, Fraser GL, Puntillo K, Ely EW, Gelinas C, Dasta JF, Davidson JE, Devlin JW, Kress JP, Joffe AM (2013). Clinical practice guidelines for the management of pain, agitation, and delirium in adult patients in the intensive care unit. Crit Care Med.

[18] Sottile PD, Albers D, Higgins C, McKeehan J, Moss MM (2018). The association between ventilator dyssynchrony, delivered tidal volume, and sedation using a novel automated ventilator dyssynchrony detection algorithm. Crit Care Med.

[19] Aragon RE, Proano A, Mongilardi N, de Ferrari A, Herrera P, Roldan R, Paz E, Jaymez AA, Chirinos E, Portugal J (2019). Sedation practices and clinical outcomes in mechanically ventilated patients in a prospective multicenter cohort. Crit Care.

[20] Conti G, Ranieri VM, Costa R, Garratt C, Wighton A, Spinazzola G, Urbino R, Mascia L, Ferrone G, Pohjanjousi P (2016). Effects of dexmedetomidine and propofol on patient-ventilator interaction in difficult-to-wean, mechanically ventilated patients: a prospective, open-label, randomised, multicentre study. Crit Care.

[21] Richman PS, Baram D, Varela M, Glass PS (2006). Sedation during mechanical ventilation: a trial of benzodiazepine and opiate in combination. Crit Care Med.

[22] Murias G, Montanya J, Chacon E, Estruga A, Subira C, Fernandez R, Sales B, de Haro C, Lopez-Aguilar J, Lucangelo U (2016). Automatic detection of ventilatory modes during invasive mechanical ventilation. Crit Care.

[23] Celis-Rodriguez E, Besso J, Birchenall C, de la Cal MA, Carrillo R, Castorena G, Ceraso D, Duenas C, Gil F, Jimenez E, et al. Clinical practice guideline based on the evidence for the management of sedoanalgesia in the critically ill adult patient. Med Intensiva. 2007;31(8):428–71. 10.1016/s0210-5691(07)74853-2 17988592

[24] Blanch L, Sales B, Montanya J, Lucangelo U, Garcia-Esquirol O, Villagra A, Chacon E, Estruga A, Borelli M, Burgueno MJ (2012). Validation of the Better Care(R) system to detect ineffective efforts during expiration in mechanically ventilated patients: a pilot study. Intensive Care Med.

[25] Bates D, Maechler M, Bolker B, Walker S (2015). Fitting linear mixed-effects models using lme4. J Stat Softw.

[26] Stephens RJ, Ablordeppey E, Drewry AM, Palmer C, Wessman BT, Mohr NM, Roberts BW, Liang SY, Kollef MH, Fuller BM (2017). Analgosedation practices and the impact of sedation depth on clinical outcomes among patients requiring mechanical ventilation in the ED: a cohort study. Chest.

[27] Woods JC, Mion LC, Connor JT, Viray F, Jahan L, Huber C, McHugh R, Gonzales JP, Stoller JK, Arroliga AC (2004). Severe agitation among ventilated medical intensive care unit patients: frequency, characteristics and outcomes. Intensive Care Med.

[28] Cavaliere F, Antonelli M, Arcangeli A, Conti G, Costa R, Pennisi MA, Proietti R (2002). A low-dose remifentanil infusion is well tolerated for sedation in mechanically ventilated, critically-ill patients. Can J Anaesth.

[29] Conti G, Arcangeli A, Antonelli M, Cavaliere F, Costa R, Simeoni F, Proietti R (2004). Sedation with sufentanil in patients receiving pressure support ventilation has no effects on respiration: a pilot study. Can J Anaesth.

[30] Costa R, Navalesi P, Cammarota G, Longhini F, Spinazzola G, Cipriani F, Ferrone G, Festa O, Antonelli M, Conti G (2017). Remifentanil effects on respiratory drive and timing during pressure support ventilation and neurally adjusted ventilatory assist. Respir Physiol Neurobiol.

[31] Rue M, Andrinopoulou ER, Alvares D, Armero C, Forte A, Blanch L (2017). Bayesian joint modeling of bivariate longitudinal and competing risks data: an application to study patient-ventilator asynchronies in critical care patients. Biom J.

[32] Marchuk Y, Magrans R, Sales B, Montanya J, Lopez-Aguilar J, de Haro C, Goma G, Subira C, Fernandez R, Kacmarek RM (2018). Predicting patient-ventilator asynchronies with hidden Markov models. Sci Rep.

[33] de Haro C, Lopez-Aguilar J, Magrans R, Montanya J, Fernandez-Gonzalo S, Turon M, Goma G, Chacon E, Albaiceta GM, Fernandez R (2018). Double cycling during mechanical ventilation: frequency, mechanisms, and physiologic implications. Crit Care Med.

[34] Dakour-Aridi H, Malas MB (2018). Less biased estimation of the survival benefit of carotid endarterectomy using real-world data: bridging the gap between observational studies and randomized clinical trials. JAMA Netw Open.

[35] Corrigan-Curay J, Sacks L, Woodcock J (2018). Real-world evidence and real-world data for evaluating drug safety and effectiveness. JAMA.

[36] Sherman RE, Anderson SA, Dal Pan GJ, Gray GW, Gross T, Hunter NL, LaVange L, Marinac-Dabic D, Marks PW, Robb MA (2016). Real-world evidence - what is it and what can it tell us?. N Engl J Med.

[37] Vincent JL, Shehabi Y, Walsh TS, Pandharipande PP, Ball JA, Spronk P, Longrois D, Strom T, Conti G, Funk GC (2016). Comfort and patient-centred care without excessive sedation: the eCASH concept. Intensive Care Med.

[38] Bauer TM, Ritz R, Haberthur C, Ha HR, Hunkeler W, Sleight AJ, Scollo-Lavizzari G, Haefeli WE (1995). Prolonged sedation due to accumulation of conjugated metabolites of midazolam. Lancet.

