# Supplementary material for: Effects of sedatives and opioids on trigger and cycling asynchronies throughout mechanical ventilation: an observational study in a large dataset from critically ill patients
Source: Crit Care. 2019 Jul 5;23:245. doi: 10.1186/s13054-019-2531-5 (PMC6612107; doi:10.1186/s13054-019-2531-5)
Supplement: Supplementary file 1 — Patient-ventilator asynchronies. (DOCX 278 kb) [file 13054_2019_2531_MOESM1_ESM.docx]

**Additional file 1. Patient-ventilator asynchronies**

Briefly, patient-ventilator asynchrony occurs when the phases of breath delivered by the ventilator do not match those of the patient. To meet the patient’s demands, the ventilator’s inspiratory time and gas delivery must match the patient’s neural inspiratory time [E1]. There are different types of patient-ventilator asynchronies. Among the most prevalent are ineffective inspiratory efforts, double cycling (DC), short cycle, and prolonged cycle. Figure E1 shows some common forms of patient-ventilator asynchronies.

Asynchronies were detected by BetterCare™ software (Barcelona, Spain), which continuously records airflow, airway pressure, and tidal volume from admission to extubation or death. The software computed the asynchrony index (AI), defined as the number of asynchronous events described above divided by the total number of ventilator cycles (machine- or patient-triggered) plus the total number of IEE multiplied by 100 [E2].

***Double cycling***

Double cycling, also named double triggering or breath stacking, consists of a sustained inspiratory effort that persists beyond the ventilator's inspiratory time, triggering a second ventilator breath, which may or may not be followed by a short expiration, where all or part of the volume of the first breath is added to the second breath [E3]. The resulting larger-than-expected tidal volume could cause ventilator-induced lung injury [E4-E6].

Detection of DC was based on previously used mathematical calculations [E3, E6-E7]. The system Better Care™ identifies DC when 1) expiratory time is ≥ 50% shorter than the averaged inspiratory time or 2) when two consecutive inspiratory cycles (positive flow –zero flow–positive flow) are detected with no expiration (negative flow) before the second inspiratory time. Additional information on factors influencing DC and its physiological implications are given in de Haro et al. [E8].

***Ineffective inspiratory efforts***

Ineffective inspiratory efforts are contractions of the inspiratory muscles, primarily the diaphragm, not followed by a ventilator breath. This asynchrony occurs when the patient’s attempt to initiate a breath does not reach the ventilator’s trigger threshold; physiologically, it is characterized by an increase in transdiaphragmatic pressure (decrease in esophageal pressure, increase in gastric pressure) and/or electrical activity of the diaphragm [E2-E9]. Ineffective inspiratory efforts result in the patient’s respiratory rate being higher than the ventilator’s rate; ineffective efforts usually occur during expiration (IEE), but can also occur during the inspiratory phase.

To detect IEE, Better Care™ computes a theoretical mono-exponential expiratory flow curve and compares it with the actual ones by evaluating its percentage deviation (0%=no deviation; 100%=maximum deviation). The theoretical curve results from the averaging of the 20 previous normal expirations in which there are no deviations that could represent an IEE. See Blanch et al. for additional information [E2].

***Short and prolonged cycle***

Short cycle is defined as an inspiratory time less than one-half the mean inspiratory time, whereas prolonged cycle occurs when an inspiratory time is greater than twice the mean inspiratory time [E3] The inspiratory time is defined as the time during which gas flow is positive, and mean inspiratory time is calculated over the previous 20 cycles.

**Supplementary References**

E1. Subira C, de Haro C, Magrans R, et al. Minimizing Asynchronies in Mechanical Ventilation: Current and Future Trends. *Respir Care* 2018;63(4):464-478.

E2. Blanch L, Sales B, Montanya J, et al. Validation of the Better Care(R) system to detect ineffective efforts during expiration in mechanically ventilated patients: a pilot study. *Intensive Care Med* 2012;38(5):772-780.

E3. Thille AW, Rodriguez P, Cabello B, et al. Patient-ventilator asynchrony during assisted mechanical ventilation. *Intensive Care Med* 2006;32(10):1515-1522.

E4. Figueroa-Casas JB, Montoya R. Effect of Tidal Volume Size and Its Delivery Mode on Patient-Ventilator Dyssynchrony. *Ann Am Thorac Soc* 2016;13(12):2207-2214.

E5. Gattinoni L, Tonetti T, Cressoni M, et al. Ventilator-related causes of lung injury: the mechanical power. *Intensive Care Med* 2016;42(10):1567-1575.

E6. Pohlman MC, McCallister KE, Schweickert WD, et al. Excessive tidal volume from breath stacking during lung-protective ventilation for acute lung injury. Crit Care Med 2008;36(11):3019-3023.

E7. Blanch L, Villagra A, Sales B, et al. Asynchronies during mechanical ventilation are associated with mortality. *Intensive Care Med* 2015;41(4):633-641.

E8. de Haro C, Lopez-Aguilar J, Magrans R, et al. Double Cycling During Mechanical Ventilation: Frequency, Mechanisms, and Physiologic Implications. *Crit Care Med* 2018;46(9):1385-1392.

E9. Georgopoulos D, Prinianakis G, Kondili E. Bedside waveforms interpretation as a tool to identify patient-ventilator asynchronies. *Intensive Care Med* 2006;32(1):34-47.

**Figure E1.** Example of some common forms of patient-ventilator asynchronies. Capture of Better Care^TM^ software showing tracing of airflow, airway pressure and volume. Episodes of double cycling (red shaded areas) and ineffective efforts during expiration (red arrows) were identified by Better Care™ (red marks bottom).
