# Supplementary material for: Effects of sedatives and opioids on trigger and cycling asynchronies throughout mechanical ventilation: an observational study in a large dataset from critically ill patients
Source: Crit Care. 2019 Jul 5;23:245. doi: 10.1186/s13054-019-2531-5 (PMC6612107; doi:10.1186/s13054-019-2531-5)
Supplement: Supplementary file 2 — Asynchronies and medication dose and asynchronies and medication dose plus SOFA as a potential confounding variable. (DOCX 86 kb) [file 13054_2019_2531_MOESM2_ESM.docx]

**Additional file 2. Asynchronies and medication dose and asynchronies and medication dose plus SOFA as a potential confounding variable**

**Table S1.** Mean estimated effect from the regression coefficients of medication dose on asynchronies, by treatment group.

| **TG:DE** | **Asynchrony Index** | **Ineffective inspiratory efforts during expiration** | **Double cycling** |
| --- | --- | --- | --- |
| **Regression coefficients** | | | |
| TG1:DE1 | -0.081 (-0.164, 0.003)  p = 0.058 | -0.015 (-0.112, 0.082)  p = 0.762 | -0.200 (-0.289, -0.111)  p < 0.0001 |
| TG3:DE1 | 0.090 (0.040, 0.139)  p = 0.0004 | 0.137 (0.080, 0.195)  p < 0.0001 | 0.037 (-0.005, 0.078)  p = 0.082 |
| TG2:DE2 | -1.130 (-1.682, -0.577)  p < 0.001 | -1.166 (-1.782, -0.550)  p = 0.0002 | -0.662 (-0.735, -0.590)  p < 0.0001 |
| TG3:DE2 | -0.565 (-0.801, -0.328)  p < 0.0001 | -0.685 (-0.960, -0.411)  p < 0.0001 | -0.485 (-0.554, -0.417)  p < 0.0001 |
| **Model’s structure and performance** | | | |
| Generalized linear mixed model fit by maximum likelihood (Laplace Approximation) ['glmerMod']  Family: Negative Binomial ( log )  Formula: AsinCount ~ TG + TG:DE1 + TG:DE2 + (1 \| Id)  Offset: log (Totalbreath + IEE) | | | |
| AIC | 6733.0 | 6273.6 | 5289.8 |
| BIC | 6769.6 | 6310.2 | 5326.5 |
| Loglik | -3357.5 | -3127.8 | -2635.9 |

Results are expressed as mean estimated effect and 95% CI

A negative sign indicates an inverse association.

TG Treatment group (TG1 sedatives-only, TG2 opioids-only, TG3 sedatives-plus-opioids)

DE Dose-equivalents (DE1 midazolam dose-equivalents, DE2 morphine dose-equivalents)

AsinCount count of asynchronies for AI, IEE, or DC each day

Id Subject factor

Totalbreath number of ventilator-delivered cycles each day

AIC Akaike information criterion

BIC Bayesian information criterion

Loglik Log likelihood

**Table S2.** Mean estimated effect from the regression coefficients of medication dose and SOFA on asynchronies, by treatment group.

| **TG:DE**  **and**  **TG:SOFA** | **Asynchrony Index** | **Ineffective inspiratory efforts during expiration** | **Double cycling** |
| --- | --- | --- | --- |
| **Regression coefficients** | | | |
| TG1:DE1 | -0.074 (-0.160, 0.012)  p = 0.090 | -0.009 (-0.109, 0.090)  p = 0.854 | -0.189 (-0.280, -0.098)  p < 0.0001 |
| TG3:DE1 | 0.089 (0.039, 0.138)  p = 0.0005 | 0.137 (0.079, 0.195)  p < 0.0001 | 0.037 (-0.021, 0.095)  p = 0.208 |
| TG2:DE2 | -1.032 (-1.627, -0.437)  p = 0.0007 | -1.048 (-1.709, -0.387)  p = 0.0019 | -0.370 (-1.110, 0.370)  p = 0.327 |
| TG3:DE2 | -0.549 (-0.793, -0.305)  p < 0.0001 | -0.675 (-0.960, -0.390)  p < 0.0001 | -0.481 (-0.765, -0.198)  p = 0.0009 |
| TG1:SOFA | 0.007 (-0.067, 0.080)  p = 0.861 | 0.050 (-0.034, 0.135)  p = 0.244 | -0.069 (-0.156, 0.017)  p = 0.117 |
| TG2:SOFA | -0.029 (-0.108, 0.049)  p = 0.466 | -0.033 (-0.124, 0.057)  p = 0.472 | -0.087 (-0.181, 0.007)  p = 0.069 |
| TG3:SOFA | -0.010 (-0.055, 0.035)  p = 0.654 | 0.004 (-0.050, 0.057)  p = 0.896 | -0.028 (-0.077, 0.022)  p = 0.270 |
| **Model’s structure and performance** | | | |
| Generalized linear mixed model fit by maximum likelihood (Laplace Approximation) ['glmerMod']  Family: Negative Binomial ( log )  Formula: AsinCount ~ TG + TG:DE1 + TG:DE2 + TG:SOFA + (1 \| Id)  Offset: log(Totalbreath + IEE) | | | |
| AIC | 6615.0 | 6170.9 | 5191.3 |
| BIC | 6663.6 | 6219.5 | 5239.9 |
| Loglik | -3295.5 | -3073.4 | -2583.6 |

Results are expressed as mean estimated effect and 95% CI

A negative sign indicates an inverse association.

TG Treatment group (TG1 sedatives-only, TG2 opioids-only, TG3 sedatives-plus-opioids)

DE Dose-equivalents (DE1 midazolam dose-equivalents, DE2 morphine dose-equivalents)

SOFA Sequential Organ Failure Assessment

AsinCount count of asynchronies for AI, IEE, or DC each day

Id Subject factor

Totalbreath number of ventilator-delivered cycles each day

AIC Akaike information criterion

BIC Bayesian information criterion

Loglik Log likelihood
