# Supplementary material for: Effects of sedatives and opioids on trigger and cycling asynchronies throughout mechanical ventilation: an observational study in a large dataset from critically ill patients
Source: Crit Care. 2019 Jul 5;23:245. doi: 10.1186/s13054-019-2531-5 (PMC6612107; doi:10.1186/s13054-019-2531-5)
Supplement: Supplementary file 3 — Sedatives and opioids dose and level of consciousness. (DOCX 68 kb) [file 13054_2019_2531_MOESM3_ESM.docx]

**Additional file 3. Sedatives and opioids dose and level of consciousness.**

**Figure S2.** Relationship between the dose of sedatives and opioids administered and level of consciousness. Statistically significant changes in SAS level per dose-equivalents are indicated with p-values.

**
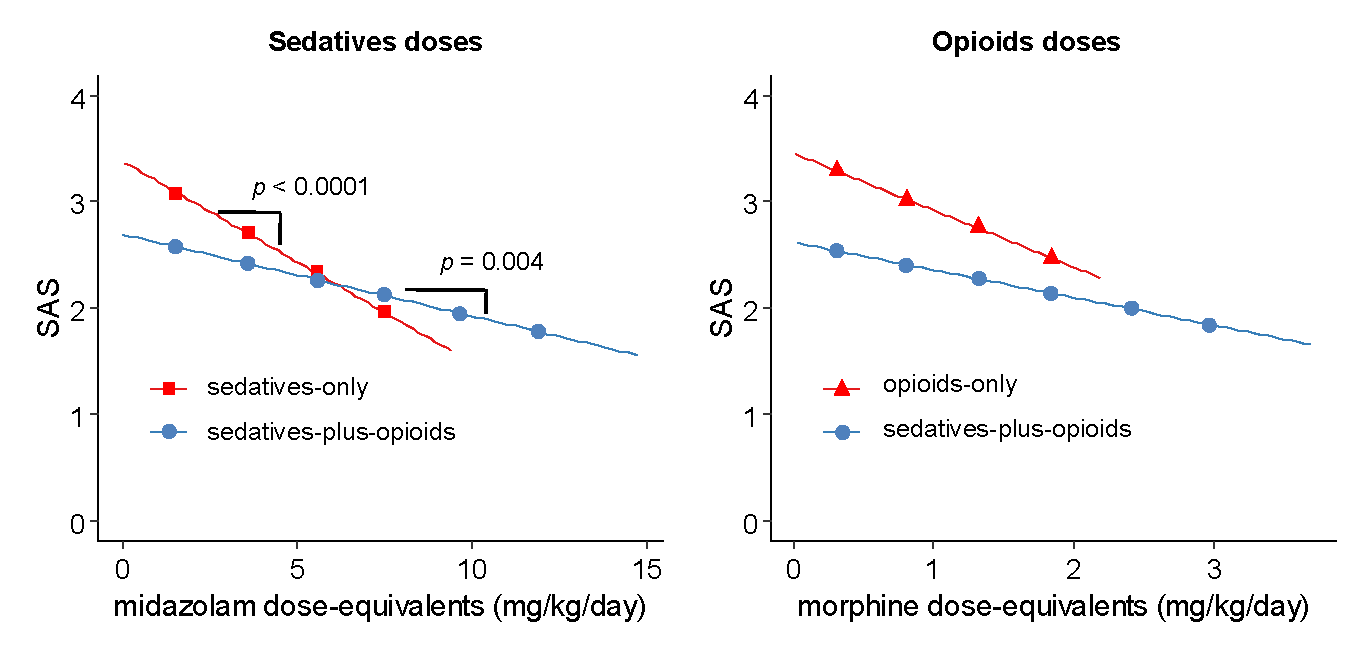
**
