# Supplementary material for: Effects of sedatives and opioids on trigger and cycling asynchronies throughout mechanical ventilation: an observational study in a large dataset from critically ill patients
Source: Crit Care. 2019 Jul 5;23:245. doi: 10.1186/s13054-019-2531-5 (PMC6612107; doi:10.1186/s13054-019-2531-5)
Supplement: Supplementary file 4 — Asynchronies and treatment group plus mechanical ventilation mode. (DOCX 78 kb) [file 13054_2019_2531_MOESM4_ESM.docx]

**Additional file 4. Asynchronies and treatment group plus mechanical ventilation mode**

| **Table S3.** Statistical significances for the comparisons between mechanical ventilation modes (AC vs. PS) in each treatment group, and among treatment groups, in each mode. | | | |
| --- | --- | --- | --- |
| **TG and MV mode** | **Asynchrony Index** | **Ineffective inspiratory efforts during expiration** | **Double cycling** |
| TG0:AC vs TG0:PS | p = 0.2537 | p = 0.1812 | p = 0.4005 |
| TG1:AC vs TG1:PS | p = 0.0348 | p = 0.0246 | p = 0. 8634 |
| TG2:AC vs TG2:PS | p = 0.2080 | p = 0.1263 | p = 0.7744 |
| TG3:AC vs TG3:PS | p = 0.0527 | p = 0.0156 | p = 0.6006 |
| **Assist-control modes** | | | |
| TG0:AC vs TG1:AC | p = 0.1293 | p = 0.1505 | p = 0.9417 |
| TG0:AC vs TG2:AC | **p = 0.0065** | p = 0.0135 | p = 0.5795 |
| TG0:AC vs TG3:AC | **p = 0.0028** | **p = 0.0069** | p = 0.0797 |
| TG1:AC vs TG2:AC | p = 0.3452 | p = 0.4367 | p = 0.6601 |
| TG1:AC vs TG3:AC | p = 0.3485 | p = 0.4509 | p = 0.1310 |
| TG2:AC vs TG3:AC | p = 0.7545 | p = 0.7772 | p = 0.3861 |
| **Pressure-support modes** | | | |
| TG0:PS vs TG1:PS | p = 0.4792 | p = 0.4036 | p = 0.5009 |
| TG0:PS vs TG2:PS | p = 0.1277 | p = 0.2380 | p = 0.1690 |
| TG0:PS vs TG3:PS | p = 0.4099 | p = 0.6880 | p = 0.2566 |
| TG1:PS vs TG2:PS | p = 0.1212 | p = 0.1515 | p = 0.7819 |
| TG1:PS vs TG3:PS | p = 0.2638 | p = 0.3315 | p = 0.8932 |
| TG2:PS vs TG3:PS | p = 0.5684 | p = 0.5023 | p = 0.8566 |

TG Treatment group (TG0 no drugs, TG1 sedatives-only, TG2 opioids-only, TG3 sedatives + opioids)

Mechanical ventilation modes (AC assist-control, PS pressure support)

Statistically significant p < 0.01
