# Supplementary material for: Effects of sedatives and opioids on trigger and cycling asynchronies throughout mechanical ventilation: an observational study in a large dataset from critically ill patients
Source: Crit Care. 2019 Jul 5;23:245. doi: 10.1186/s13054-019-2531-5 (PMC6612107; doi:10.1186/s13054-019-2531-5)
Supplement: Supplementary file 5 — Checking assumptions of the (generalized) linear mixed-effects models. (DOCX 369 kb) [file 13054_2019_2531_MOESM5_ESM.docx]

**Additional file 5. Checking assumptions of the (generalized) linear mixed-effect models**

When using LME models for the continuous response variables (SAS and SOFA), we checked the normality assumptions for the estimated random effects and for the within-subject residuals by graphical methods (normal Q-Q plots). When the response variable was discrete, as for the number of asynchronies, we assessed overdispersion by graphical comparison of the standardized residuals versus the fitted values.

To summarize, the assumptions of the LME and GLME models were met for the set of models developed, as is shown in the diagnostic plots, except for the LME model for the SOFA according to treatment group, where the within-subject residuals depart from the theoretical normal distribution (Figure S2 left). Results of the diagnostics for each model are shown in the following subsections.

***Normality of the LME model for the SAS level according to treatment group***

**Figure S3.** Q-Q plots of the within-subject residuals (left) and for the estimated random effects (right) of the LME model for SAS level according to treatment group.

***Normality of the LME model for the SOFA according to treatment group***

**Figure S4.** Q-Q plots of the within-subject residuals (left) and for the estimated random effects (right) of the LME model for the SOFA according to treatment group.

***Assessing overdispersion of the GLME models for the asynchrony rates according to treatment group***

**Figure S5.** Standardized residuals versus fitted values of the GLME models for the AI (top), IEE (bottom-left) and DC (bottom-right) rates according to treatment group.

***Assessing overdispersion of the GLME models for the asynchrony rates according to SAS level and treatment group, and according to SOFA and treatment group.***

**Figure S6.** Standardized residuals versus fitted values of the GLME models for the AI (top), IEE (middle), and DC (bottom) rates according to SAS level and treatment group (left), and according to SOFA and treatment group (right).

***Assessing overdispersion of the GLME models for the asynchrony rates according to medication doses and treatment group***

**Figure S7.** Standardized residuals versus fitted values of the GLME models for the AI (top), IEE (bottom-left), and DC (bottom-right) rates according to medication doses and treatment group.

***Normality of the LME model for the SAS level according to medication doses and treatment group***

**Figure S8.** Q-Q plots of the within-subject residuals (left) and for the estimated random effects (right) of the LME model for the SAS level according to medication doses and treatment group.

***Assessing overdispersion of the GLME models for the asynchrony rates according to medication doses and treatment group and SOFA***

**Figure S9.** Standardized residuals versus fitted values of the GLME models for the AI (top), IEE (bottom-left), and DC (bottom-right) rates according to medication doses and treatment group with SOFA as a potential confounding factor.

***Assessing overdispersion of the GLME models for the asynchrony rates according to ventilatory mode and treatment group.***

**Figure S10.** Standardized residuals versus fitted values of the GLME models for the AI (top), IEE (bottom-left), and DC (bottom-right) rates according to ventilatory mode and treatment group.
